# A novel privacy-preserving biometric authentication scheme

**DOI:** 10.1371/journal.pone.0286215

**Published:** 2023-05-25

**Authors:** Xuechun Mao, Ying Chen, Cong Deng, Xiaqing Zhou

**Affiliations:** 1 Department of Computer Science, Taizhou University, Taizhou, Zhejiang, China; 2 Hangzhou Jinritoutiao Technology Co., Ltd, Hangzhou, Zhejiang, China; 3 Medical Records Division, Taizhou Hospital, Taizhou, Zhejiang, China; University College of Engineering Tindivanam, INDIA

## Abstract

Most existing secure biometric authentication schemes are server-centric, and users must fully trust the server to store, process, and manage their biometric data. As a result, users’ biometric data could be leaked by outside attackers or the service provider itself. This paper first constructs the EDZKP protocol based on the inner product, which proves whether the secret value is the Euclidean distance of the secret vectors. Then, combined with the Cuproof protocol, we propose a novel user-centric biometric authentication scheme called BAZKP. In this scheme, all the biometric data remain encrypted during authentication phase, so the server will never see them directly. Meanwhile, the server can determine whether the Euclidean distance of two secret vectors is within a pre-defined threshold by calculation. Security analysis shows BAZKP satisfies completeness, soundness, and zero-knowledge. Based on BAZKP, we propose a privacy-preserving biometric authentication system, and its evaluation demonstrates that it provides reliable and secure authentication.

## Introduction

Biometric authentication has been popular in services such as access control to authenticate individuals based on their biometrics. In contrast to the passwords or tokens used in conventional authentication systems, biometric data, such as fingerprint, face, or gait, can physically authenticate an individual with high assurance. Biometric data is unique and safe for every individual. With the help of powerful computer and network technology, biometrics offer the advantages of uniqueness, permanence, and reliability.

However, biometric data is prone to diverse variations and distortions due to inherent and environmental noise. Thus, biometric authentication generally employs an error tolerance mechanism. Namely, the service provider uses a biometric vector *w* as a template stored in a central database, and then the user submits a new vector *w*′ to the service provider for authentication. The user’s authentication will succeed if *w* and *w*′ are close enough under a certain distance metric.

This biometric authentication model above is server-centric, as the service provider is responsible for securing the templates and receiving the user’s biometric information in plain text. This model has several unavoidable drawbacks. First, users must completely trust the service provider to protect their templates. However, malicious service providers may misuse the templates for profit, and security attacks may result in template leakage and violation of user privacy. Second, the templates must be recovered in plaintext for distance computation and comparison, which makes it possible for adversaries to spy on the registered or freshly submitted templates. To meet these challenges, it is necessary to propose a secure, privacy-preserving, and user-centered scheme [1, 2].

To address these issues, we propose a biometric authentication zero-knowledge proof (BAZKP) scheme that preserves the benefits of biometric authentication while enhancing its security. The BAZKP is a security, privacy-protection, and user-centered authentication scheme that combines biometric technology with a zero-knowledge proof (ZKP) protocol to authenticate users while maintaining anonymity. We also provide security proofs to show that our scheme guarantees privacy for users. Moreover, we have developed a privacy-preserving biometric authentication system to accomplish a complete life cycle of BAZKP.

### Related work

#### Zero-Knowledge Proof (ZKP)

Goldwasser et al. [3] first mentioned the ZKP in 1985, which enables a prover to convince a verifier that some statement is true without revealing anything more than the truth of the statement. Then, lots of research on ZKP protocols and many kinds of ZKP protocols were proposed, such as zk-SNARK and range proof. Maller et al. [4] proposed the first zk-SNARK with entirely succulent verification for general arithmetic circuits with SRS, known as Sonic. After that, Gabizon et al. proposed a more efficient zk-SNARK which is called PLONK [5]. To make improvements on the efficiency of zk-SNARK, Lately Bünz et al. proposed Super Sonic [6].

On the other hand, Brickell et al. [7] stated the first correlative algorithm of range proof. After that, in 1998, Chan et al. [8] proposed a kind of range proof whose security depends on modulus. It is called CTF. Using Lagrange’s four-square theorem [9], Lipmaa [10] published a proof of any range for the first time. In 2005, based on Lagrange’s four-square theorem, Groth [11] demonstrated that 4*y*+ 1 could be represented as the sum of the squares of some three integers if *y* was a non-negative integer. Bootle et al. [12] improved the efficiency of ZKP by using the inner product method and recursion. In 2018, Bünz et al. proposed a range proof scheme which is called Bulletproofs [13]. Based on these works, Deng et al. [14] presented the Cuproof, a novel range proof scheme for any range. The time, communication costs and proof size of that scheme are maintained within a constant range regardless of how large the proof interval is.

#### Biometric authentication

The two primary types of biometrics applications [15] are biometric authentication and biometric identification. The existing research mainly focused on privacy-preserving biometric identification [16, 17], in which a server database is assumed to contain the templates of the registered users and their corresponding identities. Barni et al. [16] presented a privacy preserving protocol for fingerprint-based authentication. In 2018, Zhou et al. [18] proposed a user-centric biometric authentication system that allows users to use the proposed lightweight encryption approach to encrypt their templates. To improve the performance of each biometric system, Hammad et al. [19] proposed a secure multimodal biometric system using convolution neural network (CNN) and Q-Gaussian multi-support vector machine (QG-MSVM) based on different level fusion.

#### Privacy-preserving scheme

In recent years, privacy protection has become a crucial area of research and many researchers conducting extensive studies in this field. Zhang et al. [20] proposed a new privacy-preserving biometric identification scheme, in which they introduced perturb terms in each biometric data. Zhou et al. [18] proposed a user-centric biometric authentication scheme that enabled end-users to encrypt their own templates with the proposed light-weighted encryption scheme. Lee et al. [21] presented a new biometric authentication system based on blockchain which provided a decentralized and distributed mechanism for processing biometric authentication.

In addition, Azees et al. [22] proposed an efficient anonymous authentication scheme to avoid malicious vehicles entering into the VANET. After that, in 2022, Zhou et al. [23] introduced a security-enhanced solution for VANETs, whichj can resist a signature forgery attack. Liu et al. [24] proposed a novel privacy-preserving DSSE scheme for IIoTH system, which was the first DSSE scheme designed for personal health record (PHR) files database with forward security. Yang et al. [25] proposed a novel oblivious data sharing scheme employing the designed 1-out-of-n oblivious transfer protocol to achieve an efficient location-based service for users while effectively hiding location coordinates and protecting the privacy of users and servers. Wei et al. [26] presented a privacy-preserving implicit authentication framework using users’ behavior features sensed by the mobile intelligent terminal based on the artificial intelligence methodology. An efficient affine cipher-based encryption technique is proposed by Azees et al. [27] to offer a high level of confidentiality with smaller key size compared to existing encryption techniques.

To address these security flaws, Subramani et al. [28] proposed a computationally efficient privacy-preserving anonymous authentication scheme for resource-limited WBAN. In 2022, Rajasekaran et al. [29] preserved the privacy and the anonymity of the end-users (patient/doctor) using an anonymous blockchain-based authentication scheme. To overcome IoHT imposes security challenges in maintaining patient data confidentiality and privacy, a novel blockchain-based privacy-preserving authentication scheme is proposed by Rajasekaran et al. [30] as an approach for achieving efficient authentication of the patient without the involvement of a trusted entity. Jegadeesan et al. [31] proposed a public key encryption based computationally efficient mutual authentication protocol for secure data transmission between incubator monitoring systems and doctors or administrators.

### Contributions

The main contributions of this paper are summarized as follows:

- We employ the inner product to construct the Euclidean Distance Zero-knowledge Proof (EDZKP) protocol and prove that the protocol satisfies perfect completeness, perfect special honest verifier zero-knowledge, and computational witness extended emulation.
- By combining ZKP with biometric authentication, we construct a novel biometric authentication scheme called BAZKP. In this scheme, the EDZKP protocol guarantees that the secret value *v* is the Euclidean Distance between the secret vectors **w** and **w′**, and the Cuproof protocol proves whether the secret value *v* is within the range [0, *e*]. We also provide security proof to show that our scheme guarantees privacy for users.
- Based on the proposed scheme, we present a privacy-preserving biometric authentication system enabling users to utilize their biometric data for authentication while preserving users privacy. We show its viability and operational efficacy by implementing the proposed system. The experimental data obtained from our tests show the system’s potential for real-world deployment.

## Preliminaries

Before we state our scheme, we first note some underlying tools. In this paper, A is a PPT adversary, which is a probabilistic interactive Turing machine that runs in polynomial time in the security parameter λ.

### Assumptions

**Groups of unknown order.** In order to keep the soundness of our scheme, we use the RSA group G where the order of the group is unknown. The RSA group is generated by a trusted setup.

*RSA group.* In the multiplicative group G of integers modulo *n* where *n* is the product of the large primes *p* and *q*. The hardness of computing the order of the group G is as the same as the hardness of factoring *n*.

**Assumption 1 (Discrete Log Relation Assumption)** *For all PPT adversaries* A *and for all j* ≥ 2, *there exists a negligible function μ*(λ) *such that*

$$\begin{array}{c} P[\begin{array}{c} G=\text{Setup}(1^{\lambda }),\\ g_{1},\ldots ,g_{j}\overset{\$}{\leftarrow }G;\\ a_{1},\ldots ,a_{j}\in Z_{n}\leftarrow A(g_{1},\ldots ,g_{j}) \end{array}:\begin{array}{c} \exists a_{i}\neq 0,\\ \prod_{i=1}^{j}g_{i}^{a_{i}}=1 \end{array}]⩽\mu \left(\lambda \right). \end{array}$$ (1)

As Bünz et al. [13] stated, $\prod_{i=1}^{j}g_{i}^{a_{i}}=1$ is a non-trivial discrete log relation between *g*_1_, …, *g*_*j*_. The discrete log relation assumption ensures that an adversary can’t find a non-trivial relation between randomly selected group elements. This assumption is equivalent to the discrete-log assumption when *j* ≥ 1.

**Assumption 2 (Order Assumption)** *The Order Assumption holds for* Setup *if for any efficient adversary* A *there exists a negligible function μ*(λ) *such that*

$$\begin{array}{c} P[\begin{array}{c} g_{1}\neq 1\wedge g_{1}^{a_{1}}=1: \end{array}\begin{array}{c} G\overset{\$}{\leftarrow }\text{Setup}\left(\lambda \right),\\ \left(g_{1},a_{1}\right)\overset{\$}{\leftarrow }A\left(G\right),\\ \text{where}\;a_{1}\neq 0\in Z_{n},\\ \text{and}\;g_{1}\in G \end{array}]\leq \mu \left(\lambda \right). \end{array}$$ (2)

**Lemma 1** *The Order Assumption implies the Discrete Log Relation Assumption*.

*Proof.* We show that if adversary $A_{Ord}$ breaks the Order Assumption, then we can construct $A_{DL}$ which breaks the Discrete Log Relation Assumption with overwhelming probability. To get a vector $\left(g_{1},g_{2},\ldots ,g_{j}\right)\in G^{j}$ and a vector $\left(a_{1},a_{2},\ldots ,a_{j}\right)\in Z_{n}^{j}$ such that $g_{1}^{a_{1}}\cdot g_{2}^{a_{2}}\ldots g_{n}^{a_{n}}=1$ where *g*_*i*_ ≠ 1, *a*_*i*_ ≠ 0 and *i* ∈ {1, 2, …, *j*}, we run $A_{Ord}$ for *n* times and it will outputs $g_{j}\in G$ and $a_{j}\in Z$ such that $g_{j}^{a_{j}}=1$ for *j* = 1, …, *n*. And it follows $\prod_{j=1}^{n}g_{j}^{a_{j}}=1$.

### Commitments

We adopt the following definitions from [14] for our notation.

**Definition 1 (Commitments)** *A non-interactive commitment scheme consists of a pair of probabilistic polynomial time algorithms* (Setup, Com). *The setup algorithm* pp ← Setup(1^λ^) *generates the public parameters pp with the security parameter* λ. *The commitment algorithm* Com_*pp*_ *defines a function* M_*pp*_ × R_*pp*_ → C_*pp*_ *for a message space* M_*pp*_, *a randomness space* R_*pp*_ *and a commitment space* C_*pp*_ *determined by pp*. *For a message* x ∈ M_*pp*_, *the algorithm draws* $r\overset{\$}{\leftarrow }R_{pp}$ *uniformly at random, and computes the commitment* **com** = Com_*pp*_(*x*, *r*).

**Definition 2 (Homomorphic Commitments)** *A homomorphic commitment scheme is a non-interactive commitment scheme such that* (M_*pp*_, *), (R_*pp*_, +), *and* (C_*pp*_, +) *are all abelian groups, and for all* x_1_, x_2_ ∈ M_*pp*_, r_1_, r_2_ ∈ R_*pp*_, *we have*

$$\begin{array}{c} \text{Com}\left(x_{1};r_{1}\right)*\text{Com}\left(x_{2};r_{2}\right)=\text{Com}\left(x_{1}+x_{2};r_{1}+r_{2}\right). \end{array}$$ (3)

Here (M_*pp*_, *) can be additive or multiplicative. For ease of notation, we drop *pp* in the subindex.

**Definition 3 (Hiding Commitment)** *A commitment scheme is said to be hiding if for every PPT adversary* A *there exists a negligible function* *μ*(λ) *such that*

$$\begin{array}{c} \left|\text{P}\left[\begin{array}{cc} b=b^{\prime } & \left|\begin{array}{c} pp\leftarrow \text{Setup}\left(1^{\lambda }\right);\\ \left(x_{0},x_{1}\right)\in {\text{M}}_{pp}^{2}\leftarrow A\left(pp\right),\\ b\overset{\$}{\leftarrow }\left(0,1\right),r\overset{\$}{\leftarrow }{\text{R}}_{pp},\\ com=\text{Com}\left(x_{b};r\right),\\ b^{\prime }\leftarrow A\left(pp,com\right) \end{array}\right. \end{array}\right]-\frac{1}{2}\right|\leq \mu \left(\lambda \right), \end{array}$$ (4)

*where the probability is over b, r,* Setup *and* A. *If μ*(λ) = 0 *then we say that the scheme is* perfectly hiding.

**Definition 4 (Binding Commitment)** *A commitment scheme is said to be binding if for every PPT adversary* A *there exists a negligible function μ such that*

$$\begin{array}{c} \text{P}\left[\begin{array}{cc} \begin{array}{c} \text{Com}\left(x_{0};r_{0}\right)=\text{Com}\left(x_{1};r_{1}\right),\\ x_{0}\neq x_{1} \end{array} & \left|\begin{array}{c} pp\leftarrow \text{Setup}\left(1^{\lambda }\right),\\ x_{0},x_{1},r_{0},r_{1}\leftarrow A\left(pp\right) \end{array}\right. \end{array}\right]\leq \mu \left(\lambda \right), \end{array}$$ (5)

*where the probability is over* Setup *and* A. *If μ*(λ) = 0, *then we say that the scheme is* perfectly binding.

In the following content, to ensure that the discrete log in the groups we used is intractable for PPT adversaries, the order of these groups is implicitly dependent on the security parameter.

**Definition 5 (Pedersen Commitment)** $M_{pp},R_{pp}=Z_{n}\;\text{and}\;C_{pp}=\left(G,*\right)$ *being a multiplicative group*.

$$\begin{array}{c} \text{Setup}:g,h\overset{\$}{\leftarrow }G, \end{array}$$ (6)

$$\begin{array}{c} \text{Com}\left(x;r\right)=\left(g^{x}h^{r}\right). \end{array}$$ (7)

**Definition 6 (Pedersen Vector Commitment)** $M_{pp}=Z_{n}^{j},R_{pp}=Z_{n}\;\text{and}\;C_{pp}=\left(G,*\right)$ *being a multiplicative group*.

$$\begin{array}{c} \text{Setup}:g=\left(g_{1},\ldots ,g_{j}\right),h\overset{\$}{\leftarrow }G, \end{array}$$ (8)

$$\begin{array}{c} \text{Com}\left(x=\left(x_{1},\ldots ,x_{j}\right);r\right)=h^{r}g^{x}=h^{r}\prod_{i}g_{i}^{x_{i}}\in G. \end{array}$$ (9)

The Pedersen vector commitment for the group G is perfectly hiding and computationally binding under the discrete logarithm assumption. In the definition, *r* is chosen at random.

### Zero-knowledge arguments of knowledge

In our scheme, we construct the zero-knowledge arguments of knowledge. A zero-knowledge proof of knowledge means a prover can convince a verifier that some statements hold without revealing any information of the knowledge. An argument is a proof that holds when the prover is computationally bounded and certain computational hardness assumptions hold. The formal definitions are as follows.

Zero-knowledge arguments consist of three interactive algorithms (Setup, P, V), which run in probabilistic polynomial time. Setup is the common reference string generator, P is the prover and V is the verifier. The algorithm Setup produces a common reference string *σ* on inputting 1^λ^. The transcript produced by P and V is denoted by tr←<P(s),V(t)>, when they interact on the inputs *s* and *t*. We write [P(s),V(t)]=b where *b* = 0 if verifier rejects, *b* = 1 if verifier accepts.

We let $R\subset {\left\{0,1\right\}}^{*}\times {\left\{0,1\right\}}^{*}\times {\left\{0,1\right\}}^{*}$ be a polynomial-time-decidable ternary relation. Given a parameter *σ*, the *w* is a witness for a statement *u* only if (σ,u,w)∈R. We define the CRS-dependent language

$$\begin{array}{c} L_{\sigma }=\left\{u|\exists w:\left(\sigma ,u,w\right)\in R\right\} \end{array}$$ (10)

as the set of all the statements which have a witness *w* in the relation R.

**Definition 7 (Argument of Knowledge)** *The triple* (Setup,P,V) *is called an argument of knowledge for relation R if it satisfies both the Perfect Completeness and Computational Witness-Extended Emulation as defined in* [13], *respectively*.

**Definition 8 (Perfect Completeness)** (Setup,P,V) *has perfect completeness if for all non-uniform polynomial time adversaries* A

$$\begin{array}{c} P[\begin{array}{cc} \begin{array}{c} (\sigma ,u,w)\notin R\;\\ or\;[P(\sigma ,u,w),V(\sigma ,u)]=1 \end{array}| & \begin{array}{c} \sigma \leftarrow \text{Setup}(1^{\lambda })\\ \left(u,w)\leftarrow A(\sigma \right) \end{array} \end{array}]=1. \end{array}$$ (11)

**Definition 9 (Computational Witness-Extended Emulation)** *For every deterministic polynomial time* $P^{*}$ *there exists an expected polynomial time emulator ε such that for every pair of interactive adversaries* $A_{1}\;\text{and}\;A_{2}$ , *there exists a negligible function μ*(λ)

$$\begin{array}{c} \left|\begin{array}{c} \text{P}\left[\begin{array}{cc} A_{1}\left(tr=1\right) & \left|\begin{array}{c} \sigma \leftarrow \text{Setup}\left(1^{\lambda }\right),\\ \left(u,s\right)\leftarrow A_{2}\left(\sigma \right),\\ tr\leftarrow \langle P^{*}\left(\sigma ,u,s\right),\\ V\left(\sigma ,u\right)\rangle \end{array}\right. \end{array}\right]-\\ \text{P}\left[\begin{array}{c} A_{1}\left(tr\right)=1\\ \wedge (tr\;\text{is}\;\text{accepting}\\ \Rightarrow \left(\sigma ,u,w\right)\in R) \end{array}\left|\begin{array}{c} \sigma \leftarrow \text{Setup}\left(1^{\lambda }\right),\\ \left(u,s\right)\leftarrow A_{2}\left(\sigma \right),\\ \left(tr,w\right)\leftarrow \varepsilon^{O}\left(\sigma ,u\right) \end{array}\right.\right] \end{array}\right|\leq \mu \left(\lambda \right), \end{array}$$ (12)

*where the oracle is given by* $O=\langle P^{*}\left(\sigma ,u,s\right),V\left(\sigma ,u\right)\rangle$, *and permits rewinding at each round after the prover commits and resuming with fresh randomness for the verifier from this point onwards, then we say* (Setup,P,V) *has witness-extended emulation*.

**Definition 10 (Public Coin)** *An argument of knowledge* (Setup,P,V) *is called public coin if all messages sent from the verifier to the prover are chosen uniformly at random and independent of the prover’s messages, i.e., the challenges correspond to the randomness ρ*.

**Definition 11 (Perfect Special Honest-Verifier Zero-Knowledge)** *A public coin argument of knowledge* (Setup,P,V) *is a perfect special honest verifier zero knowledge (SHVZK) argument of knowledge for* R *if there exists a probabilistic polynomial time simulator* S *such that for every pair of interactive adversaries* $A_{1}\;\text{and}\;A_{2}$ :

$$\begin{array}{c} \begin{array}{c} \text{P}\left[\begin{array}{cc} \left(\sigma ,u,w\right)\in R\;\text{and}\;A_{1}\left(tr\right)=1 & \left|\begin{array}{c} \sigma \leftarrow \text{Setup}\left(1^{\lambda }\right)\\ \left(u,w,\rho \right)\leftarrow A_{2}\left(\sigma \right),\\ tr\leftarrow \langle P\left(\sigma ,u,w\right),\\ V\left(\sigma ,u;\rho \right)\rangle \end{array}\right. \end{array}\right]\\ =\text{P}\left[\begin{array}{cc} \left(\sigma ,u,w\right)\in R\;\text{and}\;A_{1}\left(tr\right)=1 & \left|\begin{array}{c} \sigma \leftarrow \text{Setup}\left(1^{\lambda }\right),\\ \left(u,w,\rho \right)\leftarrow A_{2}\left(\sigma \right),\\ tr\leftarrow S\left(u,\rho \right) \end{array}\right. \end{array}\right] \end{array} \end{array}$$ (13)

*where ρ is the public coin randomness used by the verifier. The “transcript” can be simulated by S without knowing w*.

In this definition, the adversary chooses a distribution over statements and witnesses. However, the adversary still cannot distinguish between the simulated and the honestly generated transcripts for valid statements and witnesses.

Now we define range proofs. Range proofs are the proofs that the prover knows an opening to a commitment in which the committed value is in a certain range. Range proofs can be used to state that an integer commitment is for a positive number or when two homomorphic commitments are added together, it will not overflow when they are taken modulo the given prime, and these two homomorphic commitments are the commitments to the elements in a prime field.

**Definition 12 (Zero-Knowledge Range Proof)** *Given a commitment scheme* (Setup, Com) *over a message space* M_*pp*_ *which is a set with a total ordering, a zero-knowledge range proof is a SHVZK argument of knowledge for the relation*:

$$\begin{array}{c} R_{\text{Range}}:\left(pp,\left(\text{com},l,r\right),\left(x,\rho \right)\right)\in R_{\text{Range}}↔\text{com}=\text{Com}\left(x;\rho \right)\;\wedge \;\left(l\leq x<r\right). \end{array}$$ (14)

**Theorem 1 (Lagrange’s three-square theorem)** *If x is a positive integer, then* 4*x* + 1 *can be written as the sum of three squares*.

The proof for Theorem 1 is given in [9, 11] offered an efficient and simple algorithm for finding three such squares. Theorem 1 also means writing 4*x* + 1 as the sum of three squares implies that *x* is non-negative.

### Notation

Let [*N*] denote the set {1, …, *N*−1}. Let *p* and *q* denote two prime numbers. Let G denote the multiplicative group of integers modulo *n*, where *n* is the product of *p* and *q*, i.e. G is a RSA group. Let $Z_{n}$ denote the ring of integers modulo *n*. Let Z denote the set of all integers. Let $G^{j}$ and $Z_{n}^{j}$ be vector spaces of dimension *j* over G and $Z_{n}$, respectively. Let $Z_{n}^{*}$ denote $Z_{n}\backslash \left\{0\right\}$. Group elements which represent commitments are capitalized. For example, *C* = *g*^*a*^*h*^*α*^ is a Pedersen commitment to *a* for g,h∈G. $x\overset{\$}{\leftarrow }Z_{n}^{*}$ means the uniform sampling of an element from $Z_{n}^{*}$. In this paper, $a\in F^{j}$ is a vector with elements $a_{1},\ldots ,a_{j}\in F$. For an element $c\in Z_{n}$ and a vector $a\in Z_{n}^{j}$, we denote by $b=c\cdot a\in Z_{n}^{j}$ the vector with *b*_*i*_ = *c*⋅*a*_*i*_. For the two vectors $a,b\in F^{j}$, let $\langle a,b\rangle =\sum_{i=1}^{j}a_{i}\cdot b_{i}$ denote the inner product and $a\circ b=\left(a_{1}\cdot b_{1},\ldots ,a_{j}\cdot b_{j}\right)\in F^{j}$ the Hadamard product, respectively. We define vector polynomials $P\left(x\right)=\sum_{i=0}^{d}p_{i}\cdot x^{i}\in Z^{j}\left[x\right]$ where each coefficient **p**_**i**_ is a vector in $Z^{j}$. The inner product between two vector polynomials *l*(*x*) and *r*(*x*) is defined as

$$\begin{array}{c} \left\langle l\left(x\right),r\left(x\right)\right\rangle =\sum_{i=0}^{d}\sum_{j=0}^{i}\left\langle l_{i},r_{j}\right\rangle \cdot x^{i+j}\in Z\left[x\right] \end{array}$$ (15)

Let **a**‖**b** denote the concatenation of two vectors: if $a\in Z_{n}^{j}$ and $b\in Z_{n}^{m}$ then $a∥b\in Z_{n}^{j+m}$. For 0 ≤ *ℓ* ≤ *s*, we use Python notation to denote slices of vectors:

$$\begin{array}{c} a_{\left[:\ell \right]}=a_{\left[0:\ell \right]}=\left(a_{1},\ldots ,a_{\ell }\right)\in F^{\ell }, \end{array}$$ (16)

$$\begin{array}{c} a_{\left[\ell :\right]}=a_{\left[\ell :s\right]}=\left(a_{\ell +1},\ldots ,a_{s}\right)\in F^{s-\ell }. \end{array}$$ (17)

Let *t*(*x*) = 〈**l**(*x*), **r**(*x*)〉, then the inner product is defined such that *t*(*x*) = 〈*l*(*x*), *r*(*x*)〉 holds for all $x\in Z_{n}$. For vectors $g=\left(g_{1},\ldots ,g_{j}\right)\in G^{j}$ and $a\in Z_{n}^{j}$ we write $C=g^{a}=\prod_{i=1}^{j}g_{i}^{a_{i}}\in G$. For 1 ≤ *u* we set $\vec{u}=\left(1,2,3,\ldots ,u\right)\in Z^{u}$.

## Biometric Authentication Zero-Knowledge Proof (BAZKP) scheme

In this section, we introduce our BAZKP scheme. First, we state the EDZKP protocol and the Cuproof protocol, which are essential components of the BAZKP scheme.

### EDZKP protocol

In the EDZKP protocol, The vector **w,w′,d** is a secret vector encrypted by the prover based on its biometric data, where $w,w^{\prime }\in Z^{m}$ and **d = w′−w**. The secret value *v* is the Euclidean Distance between **w** and **w′**, i.e. *v* = 〈**d**, **d**〉. We will use the EDZKP protocol to prove the following relationship:

$$\begin{array}{c} \{\left(g,h,V\in G;\;v,r\in Z_{n};\;w,w^{\prime },d\in Z^{m}\right):\;V=h^{r}g^{v},\;\left\langle d,d\right\rangle =v,\;d=w-w^{\prime }\}. \end{array}$$ (18)

**Theorem 2 (EDZKP protocol)** *The EDZKP protocol presented in this section satisfies perfect completeness, perfect special honest verifier zero-knowledge, and computational witness extended emulation*.

*Proof.* The proof for Theorem 2 is given in S2 Appendix.

EDZKP Protocol ($V\in G;\;v\in Z_{n};\;w,w^{\prime },d\in Z^{m}$)

1.
$P\;\text{gets}\;d,w,w^{\prime },\;\text{where}\;\langle d,d\rangle =v,d=w-w^{\prime };$

2.
$P\;\text{selects}\;\alpha ,\beta ,\theta ,\phi \overset{\$}{\leftarrow }Z_{n};$

3.
$P\;\text{computes}\;D=h^{\alpha }g^{d}h^{d}\in G,W=h^{\beta }g^{w}h^{w}\in G,W^{\prime }=h^{\theta }g^{w^{\prime }}h^{w^{\prime }}\in G,K=h^{\phi }\in G;$

4.
$P\;\text{selects}\;s_{L},s_{R}\overset{\$}{\leftarrow }Z_{n}^{m}$ , $\rho \overset{\$}{\leftarrow }Z_{n};$
5.
$P\;\text{computes}\;S=h^{\rho }g^{s_{L}}h^{s_{R}}\in G;$

6.
$P\to V:D,W,W^{\prime },S,K;$

7.
$V\;\text{selects}\;y,z,c\overset{\$}{\leftarrow }Z_{n}^{*}$

8.
V→P:y,z,c;

9.
$P\;\text{selects}\;\tau_{1},\tau_{2}\overset{\$}{\leftarrow }Z_{n};$

10.
$P\;\text{computes}\;T_{i}=g^{t_{i}}h^{\tau_{i}}\in G,$ where *t*_*i*_ can be computed without knowing **l** and **r**, i.e. *t*_*i*_ is the coefficient of 〈*l*(*x*), *r*(*x*)〉, respectively.i={1,2},e=c(θ+α-β)+ϕ∈Z;
11.
$P\to V:T_{1},\;T_{2},\;e;$

12.
$V\;\text{selects}\;x\overset{\$}{\leftarrow }Z_{n}^{*};$

13.
V→P:x;

14.
$P\;\text{computes}\;l=l\left(x\right)=dz-y+s_{L}x\;\in Z^{m},\;r=r\left(x\right)=dz+y+s_{R}x\;\in Z^{m},\;\hat{t}=\langle l,r\rangle =t_{0}+t_{1}x+t_{2}x^{2}\in Z;$

15.
$P\;\text{computes}\;\tau_{x}=\tau_{2}\cdot x^{2}+\tau_{1}\cdot x+z^{2}r\;\in Z,\;\mu =\alpha z+\rho x\;\in Z;$

16.
$P\to V:\tau_{x},\;\mu ,\;\hat{t},\;l,\;r;$

17.
$V\;\text{computes}\;P=D^{z}\cdot S^{x}\cdot g^{-y}\cdot h^{y}\;\in G;$

18.
$V\;\text{checks}\;\text{these}\;\text{equations}:\;P\overset{?}{=}h^{\mu }\cdot g^{l}\cdot h^{r}\in G,\;g^{\hat{t}}h^{\tau_{x}}\overset{?}{=}V^{z^{2}}g^{-\delta (y)}\cdot T_{1}^{x}\cdot T_{2}^{x^{2}}\in G,\;\hat{t}\overset{?}{=}\langle l,r\rangle \in Z,\;W^{c}h^{e}\overset{?}{=}W^{\prime c}\cdot D^{c}\cdot K.$

### Cuproof protocol

One of the effective range proof protocols is the Cuproof [14] protocol. This protocol can maintain time, communication overhead, and proof size within a fixed range regardless of how large the proof interval is. We use the Cuproof protocol in BAZKP scheme to convince the verifier that the secret number *v* is in [*a*, *b*]. Based on Lagrange three-square theorem, we can find $a,b\in Z_{n}$ and $u=\left(u_{1},u_{2},u_{3},u_{4},u_{5},u_{6}\right)\in Z_{n}^{6}$ such that the following conditions hold

$$\begin{array}{c} \left\{ \begin{array}{c} u_{1}^{2}+u_{2}^{2}+u_{3}^{2}=4v-4a+1=v_{1}\in Z,\\ u_{4}^{2}+u_{5}^{2}+u_{6}^{2}=4b-4v+1=v_{2}\in Z. \end{array}\right. \end{array}$$ (19)

In this protocol, the $\delta \left(y\right)=\langle y,y\rangle \in Z\;\text{and}\;y\in Z^{6}$. We will prove the following relations:

$$\begin{array}{c} \left\{\left(g,h\in G,V\in G^{2}\right):V_{j}=h^{r_{j}}g^{v_{j}}\;\forall \;j\in \left\{1,2\right\},V=g^{v}h^{r}\wedge v\in \left[a,b\right]\right\} \end{array}$$ (20)

**Theorem 3 (Cuproof protocol)** *The Cuproof protocol presented in this section satisfies perfect completeness, perfect special honest verifier zero-knowledge, and computational witness extended emulation*.

*Proof.* The proof for Theorem 3 is given in [14]

Cuproof (V∈G;v,a,b∈Z):

1.
$P\;\text{computes}\;v_{1}=4v-4a+1,v_{2}=4b-4v+1\;\in Z;$

2.
$P\;\text{selects}\;\alpha \overset{\$}{\leftarrow }Z_{n};$

3.
$P\;\text{computes}\;A=h^{\alpha }\prod_{j=1}^{2}g_{\left[3(j-1):3j\right]}^{j\cdot d_{\left[3(j-1):3j\right]}}\cdot \prod_{j=1}^{2}h_{\left[3(j-1):3j\right]}^{j\cdot d_{\left[3(j-1):3j\right]}}\in G;$ ,
4.
$P\;\text{selects}\;s_{L},s_{R}\overset{\$}{\leftarrow }Z_{n}^{6}$ , $\rho \overset{\$}{\leftarrow }Z_{n};$
5.
$P\;\text{computes}\;S=h^{\rho }g^{s_{L}}h^{s_{R}}\in G;$

6.
P→V:A,S;

7.
$V\;\text{selects}\;y,z\overset{\$}{\leftarrow }Z_{n}^{*};$

8.
V→P:y,z;

9.
$P\;\text{selects}\;\tau_{3},\tau_{4}\overset{\$}{\leftarrow }Z_{n};$

10.
$P\;\text{computes}\;\text{computes}\;T_{i}=g^{t_{i}}h^{\tau_{i}}\in G$ where *t*_*i*_ can be computed without knowing **l′** and **r′**, i.e. *t*_*i*_ is the coefficient of 〈*l*(*x*), *r*(*x*)〉 respectively.$\;\;i=\left\{3,4\right\},r_{1}=4r\in Z,r_{2}=-4r\;\in Z;$
11.
$P\to V:T_{3},\;T_{4};$

12.
$V\;\text{selects}\;x\overset{\$}{\leftarrow }Z_{n}^{*};$

13.
V→P:x;

14.
$P\;\text{computes}\;l^{\prime }=z\cdot \sum_{j=1}^{2}j\cdot (0^{3(j-1)}∥d_{\left[3(j-1):3j\right]}∥0^{3(2-j)})-y+s_{L}x\in Z^{6},\;r^{\prime }=z\cdot \sum_{j=1}^{2}j\cdot (0^{3(j-1)}∥d_{\left[3(j-1):3j\right]}∥0^{3(2-j)})+y+s_{R}x\in Z^{6},\;\hat{t^{\prime }}=\langle l^{\prime },r^{\prime }\rangle \in Z;$

15.
$P\;\text{computes}\;\tau_{x}^{\prime }=\tau_{4}x^{2}+\tau_{3}x+z^{2}\sum_{j=1}^{2}j^{2}\cdot r_{j}\in Z,\;\mu^{\prime }=\alpha z+\rho x\;\in Z;$

16.
$P\to V:\tau_{x}^{\prime },\;\mu^{\prime },\;\hat{t^{\prime }},\;l^{\prime },\;r^{\prime };$

17.
$V\;\text{computes}:\;P=A^{z}\cdot S^{x}\cdot g^{-y}\cdot h^{y}\;\in G,\;V_{1}=V^{4}\cdot g^{-4a}\cdot g=g^{4v-4a+1}h^{4r}=g^{v_{1}}h^{r_{1}}\in G,\;V_{2}=g^{4b}\cdot V^{-4}\cdot g=g^{4b-4v+1}h^{-4r}=g^{v_{2}}h^{r_{2}}\in G,\;V=\left(V_{1},V_{2}\right)\;\in G^{2};$

18.
$V\;\text{checks}\;\text{these}\;\text{equations}:\;P\overset{?}{=}h^{\mu^{\prime }}\cdot g^{l^{\prime }}\cdot h^{r^{\prime }}\in G,\;g^{\hat{t^{\prime }}}h^{\tau_{x}^{\prime }}\overset{?}{=}V^{z^{2}\cdot \left(\vec{2}\circ \vec{2}\right)}\cdot g^{-\delta (y)}\cdot T_{3}^{x}\cdot T_{4}^{x^{2}}\in G,\;\hat{t^{\prime }}\overset{?}{=}\langle l^{\prime },r^{\prime }\rangle \in Z;$

### Our scheme

The main idea of BAZKP scheme is set out below. Given three vectors $w,\;w^{\prime },\;d\in Z^{m}$, the secret vectors **w** and **w′** are biometric data of P, then the vectors **w,** **w′** and **d** satisfy the following relation:

$$\begin{array}{c} w=w^{\prime }+d. \end{array}$$ (21)

The objective of BAZKP scheme is to persuade the verifier V that the secret vectors **w,w′** belong to the same person, that is, the Euclidean Distance between **w,** **w′** is smaller than *e*. Let v=〈d,d〉∈Z, *v* is the Euclidean Distance between **w** and **w′**, the goal of this scheme is to prove that the secret value *v* ∈ [0, *e*].

In detail, we will prove the following relations:

$$\begin{array}{c} \{\left(v\in Z;w,w^{\prime },d\in Z^{m};g,h,V\in G\right):V=g^{v}h^{r},w=w^{\prime }+d,v=\left\langle d,d\right\rangle ,v\in \left[0,e\right]\}. \end{array}$$ (22)

First, we use Pedersen Commitment and Pedersen Vector Commitment to commit *v*, **w,w′,d**. Then, let P,V run the EDZKP protocol and the Cuproof protocol to prove the equation **w** = **w′**+ **d**, *v* = 〈**d**, **d**〉, *v* ∈ [0, *e*]. The V outputs “accept” only when the two protocols are ran successfully.

BAZKP scheme ($V\in G;\;v,\;0,\;e\in Z;\;w,w^{\prime },d\in Z^{m}$)

1.
$P\;\text{computes}\;V=g^{v}h^{r}\in G\;\text{and}\;\text{sends}\;V\;\text{to}\;V;$

2.
P,Vrun EDZKP protocol $(V\in G;\;v\in Z;\;w,w^{\prime },d\in Z^{m}).$
3.
P,VrunCuproofprotocol(V∈G;v,0,e∈Z).

4.
Voutputs“accept”onlywhenthattwoprotocolsareransuccessfully.

**Theorem 4 (BAZKP Scheme)** *The BAZKP scheme presented in this section satisfies perfect completeness, perfect special honest verifier zero-knowledge, and computational witness extended emulation*.

*Proof.* Because the EDZKP protocol and the Cuproof protocol satisfy perfect completeness, perfect special honest verifier zero-knowledge, and computational witness extended emulation. Therefore, our BAZKP scheme has perfect completeness, perfect special honest verifier zero-knowledge, and computational witness extended emulation.

This scheme can also be transformed into a NIZK scheme by using the Fiat-Shamir heuristic. For example, we set *y* = *Hash*(*D*‖*W*),*z* = *Hash*(*D*‖*W*′),*c* = *Hash*(*D*‖*S*),*x* = *Hash*(*D*‖*K*) in EDZKP protocol. And we set *y* = *Hash*(*A*‖*S*), *z* = *Hash*(*τ*_*x*_||*μ*), *x* = *Hash*(*T*1‖*T*2) in Cuproof protocol.

## Privacy-preserving biometric authentication system

In this section, we construct a privacy-preserving biometric authentication system based on BAZKP scheme and evaluate its comprehensive performance using fingerprints. Before anything else, we provide some crucial background information on biometric authentication. Then, we demonstrate how to build biometric authentication systems that protect user privacy using our suggested scheme.

### Background

The first critical step in biometric authentication is efficiently transforming biometric traits into feature vectors that are easy to compute. We adopt fingerprint vectors *w* with length 299, generated from the fingerprint minutiae proposed in [32]. The conversion process consists of two sequential stages: generation of the minutia cylinder-code with the MCC SDK and the kernel principal component analysis (KPCA) transformation. Our experiments are carried out using FVC 2002 and FVC 2004. Each of these datasets has a Set A and Set B. We used Set B to generate the fixed-length vector and Set A for the verification. In the following discussion, we assume that a sensor can capture the user’s biometric trait and transform it into a fixed-length vector on the local side.

### System construction

A privacy-preserving biometric authentication system is composed of four phases: setup, registration, query and authentication.

1. **Setup**: The user output the public parameters (*g*, *h*, **g**, **h**).
2. **Registration**: The user receives the binary fingerprint vector **w** from the sensor and stores it in his database. Then, the user generates the commitment of **w** (we use *W* to represent the commitment of **w**) and sends the *W* to the cloud server. The cloud server stores the tuple (*g*, *h*, **g**, **h**, *W*).
3. **Query**: The user uses the sensor to get a new binary fingerprint vector **w′**. Then, The user generates **d** by using **d** = **w**−**w′**, where the user’s local database contains **w**. Then the user sends **d** and ($A,S,T_{1},T_{2},\tau_{x},\;\mu ,\;\hat{t},\;l,\;r,$ $T_{3},T_{4},\tau_{x}^{\prime },\;\mu^{\prime },\;\hat{t^{\prime }},\;l^{\prime },\;r^{\prime }$) to the cloud server.
4. **Authentication**: Once the cloud sever receives *W*, it finds the tuple (*W*, *g*, *h*, **g**, **h**). The server runs the BAZKP scheme. Then, the server will output an authentication result.

**Fig 1:**
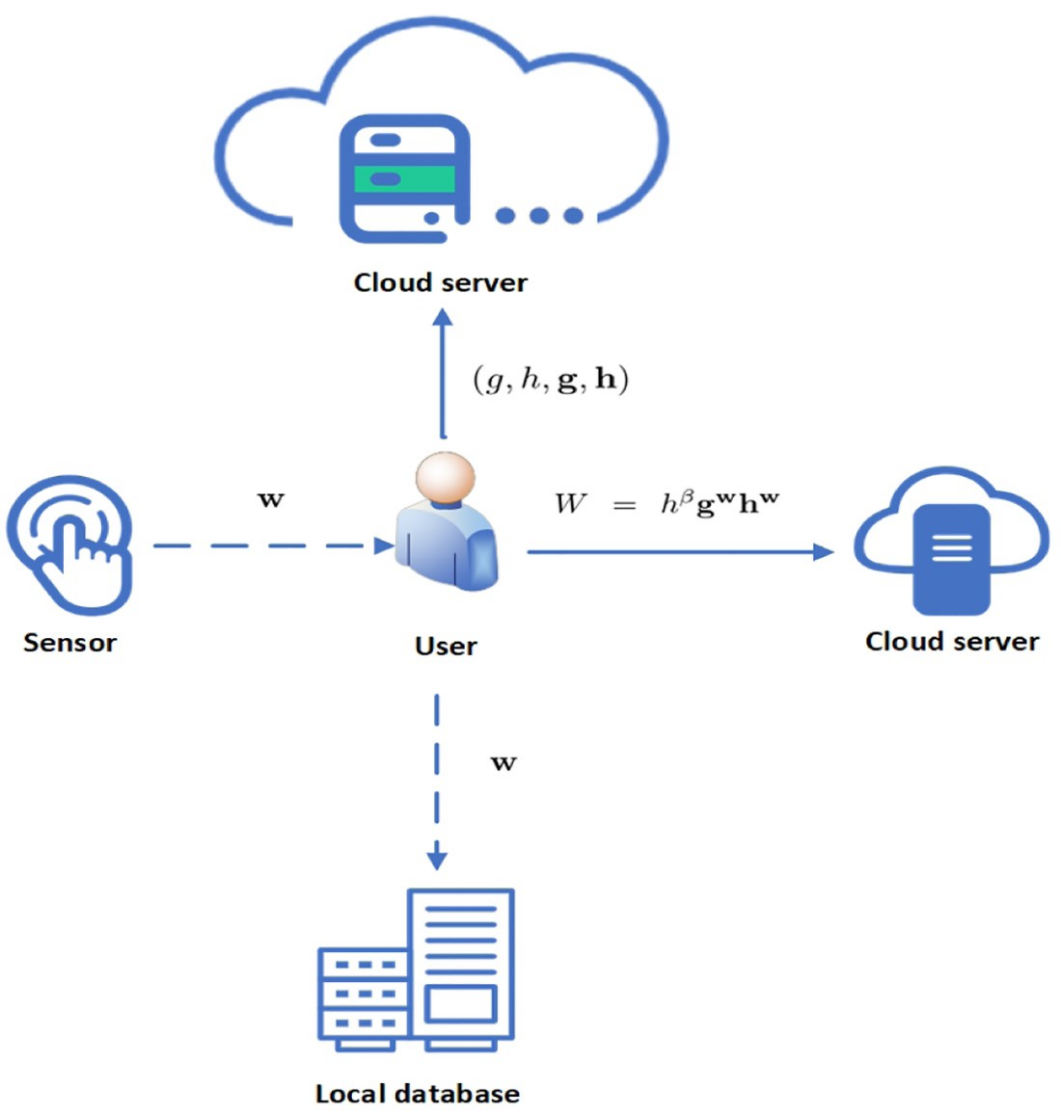
The setup and registration phase.

**Fig 2:**
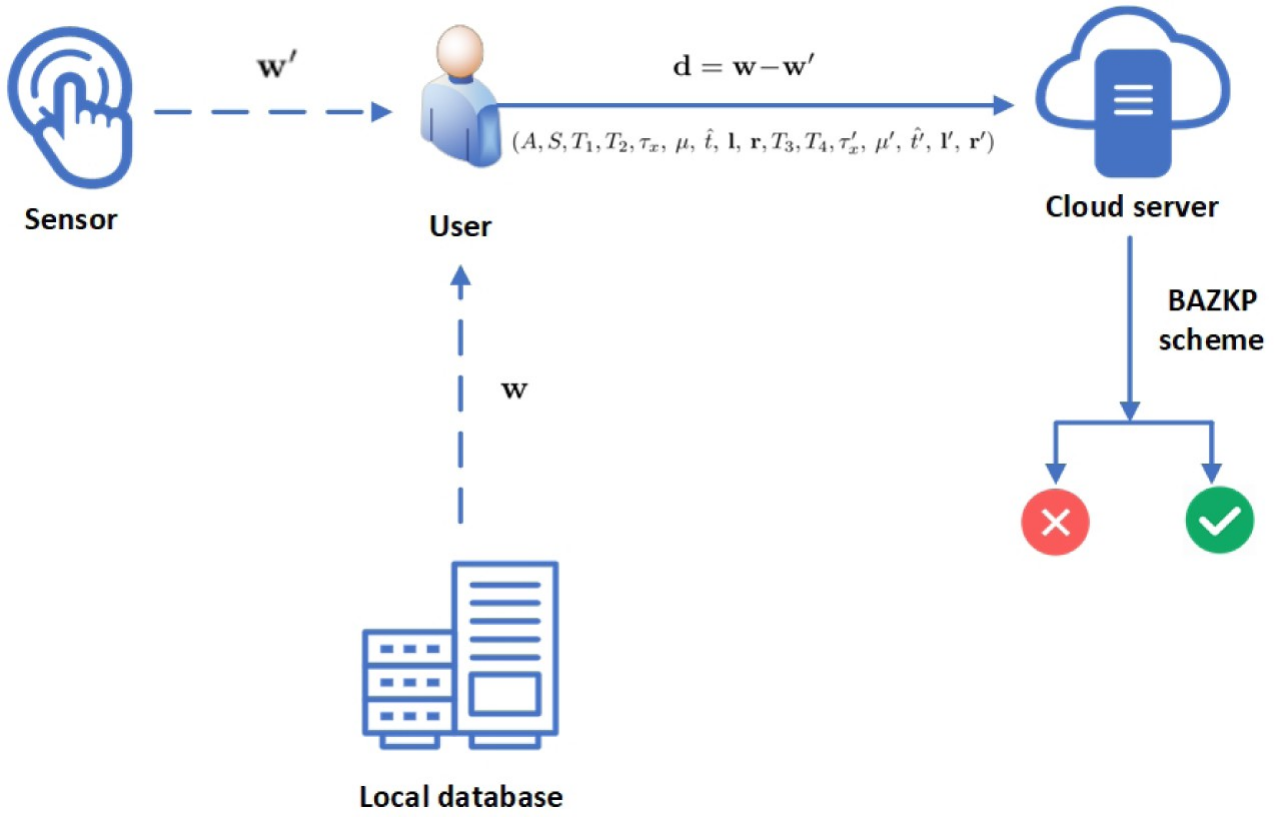
The query and authentication phase.

Fig 1 shows the setup and registration phase, Fig 2 shows the query and authentication phase.

### Security analysis

In this section, we briefly analyse the security strength of our proposed system with respect to various security attacks. First, we prove that the BAZKP scheme satisfies perfect completeness, perfect special honest verifier zero-knowledge, and computational witness extended emulation. As a result, our proposed system based on BAZKP scheme also satisfies these properties. Referring to the security analysis in [27–31], we prove that our proposed system can effectively withstand various types of attacks, including replay attacks, man in the middle attacks, impersonation attacks, and message modification attacks. Additionally, our system also supports non-traceability, further enhancing its security capabilities.

#### Replay attack and man in the middle attack

In our proposed system, the cloud server receives the tuple (*W*, *g*, *h*, **g**, **h**) before authenticating the user’s identity. Once the data is received, the cloud server maps it to the timestamp and user identity and stores them in the database. This approach allows the cloud server to defend against replay attacks by verifying if the mapped data in the database matches the data sent by the user and if the time difference between them falls within a legal interval.

Additionally, the proposed system enables mutual authentication to be performed securely between the user and the cloud server, ensuring the system can withstand man in the middle attacks.

#### Impersonation attack and message modification attack

Suppose a malicious attacker sends the tuple ($A,S,T_{1},T_{2},\tau_{x},\;\mu ,\;\hat{t},\;l,\;r,T_{3},T_{4},\tau_{x}^{\prime },\;\mu^{\prime },\;\hat{t^{\prime }},\;l^{\prime },\;r^{\prime },W,W^{\prime }$) to the cloud server where $W^{\prime }\neq h^{\theta }g^{w^{\prime }}h^{w^{\prime }}$. Once the attacker pass the authentication with overwhelming probability then it means the BAZKP scheme can not meet soundness. Since we have proven that the BAZKP scheme meets soundness, a message modification attack is impossible in the proposed system. Therefore our proposed system can withstand the message modification attack and impersonation attack.

#### Non-traceability

In this proposed system, we utilize Pedersen Vector Commitment to preserve the secret values, specifically *W* = *h*^*θ*^**g**^**w**^**h**^**w**^, based on the hardness of the DL assumption. Consider a scenario where an attacker has captured the value **w** from *W*. However, it is impossible for the attacker to compute **w** from *W* due to the difficulty of solving the DL assumption. Additionally, the user selects random numbers $\alpha ,\beta ,\theta ,\phi \in Z_{n}$ for each execution in our proposed system, and the BAZKP scheme also meets zero-knowledge. Therefore, our scheme supports non-traceability.

## Implementation and performance analysis

In this section, we present the results of our evaluation and prototype implementation. Then, compare it with other existing related biometric authentication schemes.

### Implementation

As a user-centric biometric authentication system, our system’s performance is evaluated on a personal laptop and a mobile phone. In the simulation, we utilize a mobile phone with Android 13 operating system, 3123 MHz CPU, and 8 GB RAM. We also use a personal laptop with macOS 11.2.2, Apple M1, and 8 GB RAM. The Java library UJMP and the C++ library Armadillo are used for server emulation of mobile phones and computers, respectively. At the same time, the client is written using React.

We noticed that performance depends on many factors. The system may not operate at its best due to the above choice. The size of the two primes *p*, *q* is set to 1024 bits. A Pedersen hash function over an RSA group whose modulo *n* = p*q is benchmarked. We generate witness refer to literature [9, 10]. We also set *e* = 7000, in other words, the user can pass the identity authentication only when the Euclidean distance between **w** and **w′** is less than or equal to 4.

**Fig 3:**
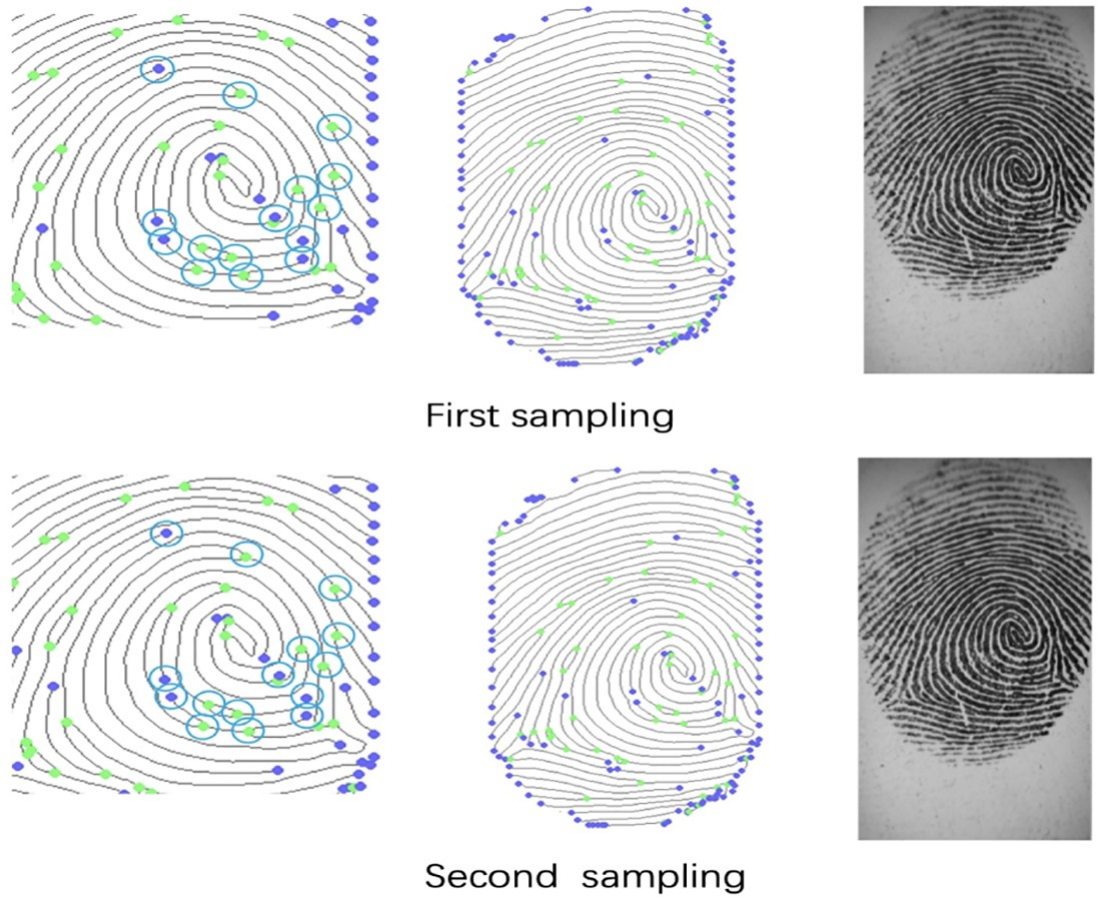
Details of fingerprint sampling.

The system uses sensors to capture the user’s fingerprints and generate the corresponding vectors. The sensor captures the fingerprint feature points and generates the fingerprint vectors based on the horizontal and vertical coordinates of these fingerprint points, as shown in Fig 3.

The final data is the average of the data we obtained by doing 10000 experiments. It is possible to conclude that our scheme’s communication cost is equal to $11G+7Z_{n}+\left(2m+19\right)Z$ through computation and analysis, indicating that it is kept constant.

**Fig 4:**
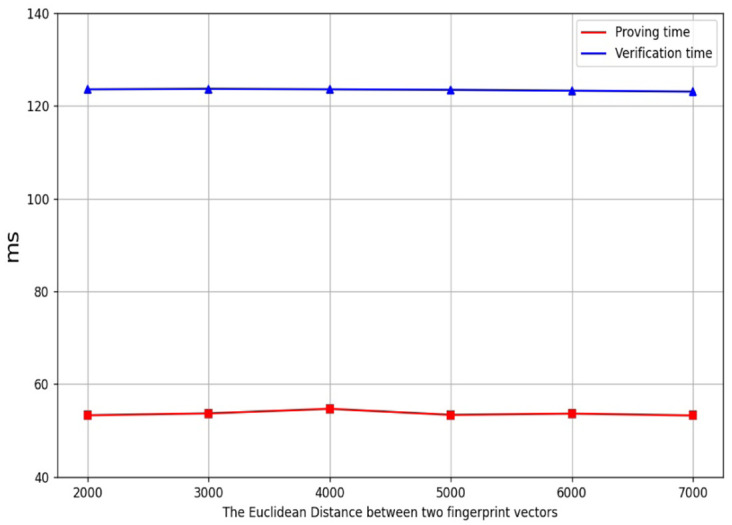
The time cost of EDZKP protocol.

**Fig 5:**
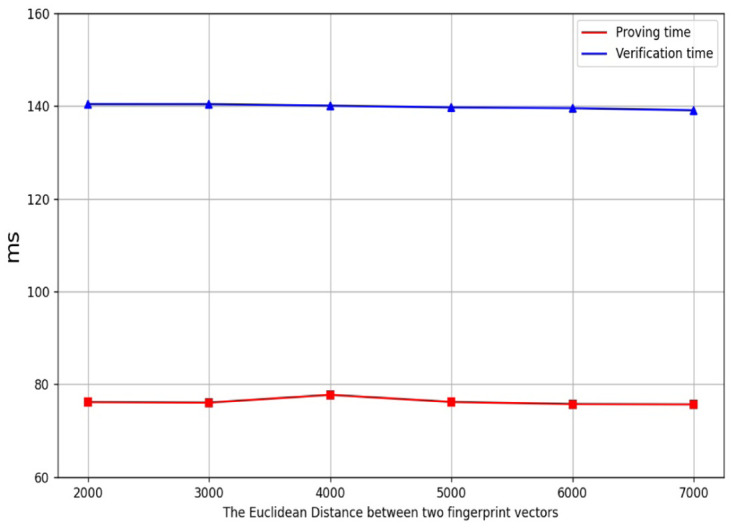
The time cost of BAZKP scheme.

Fig 4 shows the line charts of the proving time and the verification time of the EDZKP protocol, respectively. Fig 5 shows the line charts of the proving time and the verification time of the BAZKP scheme (not including witness generation), respectively. Table 1 shows our system’s proving time and verification time under the different Euclidean distance. The simulation results show that the proving and verification time of the BAZKP scheme remains almost constant with the increase of the Euclidean distance. From this, we can conclude that no matter how extensive the range is, the proving time and the verification time remain almost unchanged.

**Table 1:** BAZKP Performance.

Length of biometric vector	Euclidean distance	Timing (ms)	
		Prove	Verify
299	2000	76.14	140.44
299	3000	76.03	140.43
299	4000	77.70	140.11
299	5000	76.17	139.71
299	6000	75.70	139.57
299	7000	75.64	139.10

### Performance analysis

Finally, we compare the BAZKP scheme with other similar schemes. Table 2 shows the comparison results between our scheme, literature [20], literature [18], and literature [21]. Lee et al. [21] proposed a biometric authentication scheme that divides the biometric template into fragments and stores them on the blockchain. However, compared with our scheme, the real-time performance of this scheme is low. Similarly, Zhou et al. [18] presented a user-centric biometric authentication scheme, but it encountered an exponential increase in time complexity with the increase in *n*. In contrast, our solution offers a significant advantage regarding time complexity. Compared with the biometric authentication scheme in [20], our scheme achieves the goal of privacy protection while improving efficiency. In addition, the security of this scheme is based on the RSA assumption and the discrete log assumption, which is relatively secure compared to other schemes.

**Table 2:** Comparison results of similar schemes.

Schemes	Universal	Untrusted Setup	Assumption	Time Complexity
Literature [20]	•	∘	−	*O*(*n*^2^)
Literature [18]	•	∘	−	*O*(*n*^2^)
Literature [21]	∘	∘	−	*O*(*n*)
Our scheme	•	∘	RSA+DL	*O*(*n*)

Here *n* represents the length of the fingerprint vector. A black circle for a universal denotes that the scheme can be promoted, and a white circle for an untrusted setup denotes that the scheme is updatable. DL stands for discrete log.

## Conclusions and future work

In this paper, we combine biometrics with ZKP protocol to introduce a biometric authentication scheme for privacy-preserving named BAZKP. The security analysis shows that the scheme satisfies the perfect completeness, perfect special honest verifier zero-knowledge, and computational witness extended emulation. The evaluation results show that, no matter how big the interval of the range proof is, the proof time and the verification time are nearly constant. Finally, based on the BAZKP scheme, we build a user-centric biometric authentication system.

We will focus on improving the efficiency and safety of the BAZKP scheme in the future. One approach will be to leverage a more efficient proof-of-zero algorithm to refine the accuracy of biometric templates while preserving privacy. Another approach will be to employ multi-factor authentication, which combines biometric data with other authentication methods, such as passwords or tokens, to boost security. Additionally, combining the BAZKP scheme with blockchain technology can provide additional security and transparency to the authentication process.

## Supporting information

S1 Data (ZIP)

S1 Appendix (PDF)

S2 Appendix (PDF)
