# Supplementary material for: A novel privacy-preserving biometric authentication scheme
Source: PLoS One. 2023 May 25;18(5):e0286215. doi: 10.1371/journal.pone.0286215 (PMC10212112; doi:10.1371/journal.pone.0286215)
Supplement: S1 Appendix — (PDF) [file pone.0286215.s002.pdf]

S1 Appendix A General Forking Lemma. Maecenas convallis mauris sit amet sem ultrices gravida. Etiam eget sapien nibh. Sed ac ipsum eget enim egestas ullamcorper nec euismod ligula. Curabitur fringilla pulvinar lectus consectetur pellentesque. Now we briefly describe the forking lemma of [1] that will be needed in the proofs.

Suppose that we have a $(2\mu + 1)$-move public-coin argument with μ challenges $x_1, ..., x_\mu$ in sequence. Let $k_i\ (\geq 1) \in \mathbb{Z}$ for $1 \leq i \leq \mu$. Consider there are $\prod_{i=1}^{\mu} k_i$ accepting transcripts with challenges in the following tree format. The tree has $\prod_{i=1}^{\mu} k_i$ leaves and depth μ. The root of the tree is labeled with the statement. Each node of depth $i < \mu$ has exactly k_i children nodes, each child node is labeled with a distinct value of the ith challenge x_i.

The above structure can be referred to a $(k_1, ..., k_\mu)$-tree of accepting transcripts. Given a suitable tree of accepting transcripts, one can compute a valid witness for our inner-product argument, range proof, and argument for arithmetic circuit satisfiability. This is a natural generalization of special-soundness for Sigma-protocols with $\mu = 1$ and $k_1 = 2$. Combined with Theorem 3, this shows that the protocols have a witness-extended emulation, and hence, the prover cannot produce an accepting transcript unless they know a witness. For simplicity in the following lemma, we assume that the challenges are chosen uniformly from $\mathbb{Z}_n$ with $|n| = \lambda$, but any sufficiently large challenge space is enough. The success probability of a cheating prover scales inversely with the size of the challenge space and linearly with the number of accepting transcripts that an extractor needs. Therefore, if $\prod_{i=1}^{\mu} k_i$ is negligible in 2^λ, then a cheating prover can create a proof that the verifier accepts with only negligible probability.

Theorem 5 (Forking Lemma [1]) *Let* (Setup, $\mathcal{P}$, $\mathcal{V}$) *be a* $(2k + 1)$*-move, public coin interactive protocol. Let* χ *be a witness extraction algorithm that succeeds with probability* $1 - \mu(\lambda)$ *for some negligible function* $\mu(\lambda)$ *in extracting a witness from an* $(k_1, ..., k_k)$*-tree of accepting transcripts in probabilistic polynomial time. Assume that* $\prod_{i=1}^{k} k_i$ *is bounded above by a polynomial in the security parameter* λ*. Then* $(Setup, \mathcal{P}, \mathcal{V})$ *has witness-extended emulation.*

Theorem 5 is slightly different from the lemma 1 of [1]. We allow the extractor χ to fail with a negligible probability. Whenever this happens, the emulator ε also simply fails. Even with this slight modification, this lemma still holds as ε overall still only fails with negligible probability.

Reference

1. Bootle J, Cerulli A, Chaidos P. Efficient Zero-Knowledge Arguments for Arithmetic Circuits in the Discrete Log Setting. Advances in Cryptology - EUROCRYPT 2016; 2016: 327-357.
