# Supplementary material for: A novel privacy-preserving biometric authentication scheme
Source: PLoS One. 2023 May 25;18(5):e0286215. doi: 10.1371/journal.pone.0286215 (PMC10212112; doi:10.1371/journal.pone.0286215)
Supplement: S2 Appendix — (PDF) [file pone.0286215.s003.pdf]

S2 Appendix Proof of Theorem 2. *Proof.* Perfect completeness always holds as the fact that $t_0 = z^2 v - \delta(y),\ \mathbf{d} = \mathbf{w} - \mathbf{w}'$ for all valid witnesses. In order to prove perfect honest-verifier zero-knowledge, we construct a simulator that produces a distribution of proofs for a given statement
$\{(g, h, V \in \mathbb{G};\ v, r \in \mathbb{Z}_n;\ \mathbf{w}, \mathbf{w}', \mathbf{d} \in \mathbb{Z}^n) :\ V = h^r g^v,\ \langle \mathbf{d}, \mathbf{d} \rangle = v,\ \mathbf{d} = \mathbf{w} - \mathbf{w}'\}$ which is indistinguishable from valid proofs produced by an honest prover interacting with an honest verifier. All proof elements and challenges according to the randomness supplied by the adversary from their respective domains are chosen by the simulator or directly computed by the simulator. S and T_1 are computed according to the verification equations, i.e.:

$$S = (h^{-\mu} \cdot \mathbf{g}^{-\mathbf{1}-\mathbf{y}} \cdot \mathbf{h}^{\mathbf{y}-\mathbf{r}} \cdot D^z)^{-x^{-1}} \tag{23}$$

$$T_1 = (g^{-\hat{t}-\delta(y)} \cdot h^{-\tau_x} \cdot V^{z^2} \cdot T_2^{x^2})^{-x^{-1}} \tag{24}$$

According to the simulated witness $(\mathbf{l}, \mathbf{r})$ and the verifier's randomness, the simulator runs the inner-product argument. In the zero-knowledge proof, all elements are either independently randomly distributed or their relationship is completely defined by the verification equation. Because we can successfully simulate the witness, the inner product argument remains zero knowledge. Thus leaking information about witness or revealing it does not change the zero-knowledge property of the overall protocol. The simulator is efficient because it runs in time $O(\mathcal{V} + \mathcal{P}_{\text{InnerProduct}})$.

We construct an extractor χ to prove computational witness extended emulation. The extractor χ uses n different values of y, 3 different values of z, 2 different values of challenge c and 3 different values of the challenge x to run prover algorithm. It additionally invokes the extractor for the inner product argument on each of the transcripts. This results in $n \cdot 3 \cdot 2 \cdot 3 \cdot O(n^2)$ valid proof transcripts.

For each transcript, in order to extract the witnesses $\mathbf{l}$ and $\mathbf{r}$ to the inner product argument such that $h^{\mu}\mathbf{g}^{\mathbf{l}}\mathbf{h}^{\mathbf{r}} = P\ \wedge\ \langle \mathbf{l}, \mathbf{r} \rangle = \hat{t}$, the extractor χ first runs the extractor $\chi_{\text{InnerProduct}}$ for the inner-product argument. In order to compute $\rho, \alpha, \mathbf{d}, \mathbf{s}_L$ and $\mathbf{s}_R$ such that $D = h^{\alpha}\mathbf{g}^{\mathbf{d}}\mathbf{h}^{\mathbf{d}}$ and $S = h^{\rho}\mathbf{g}^{\mathbf{s}_L}\mathbf{h}^{\mathbf{s}_R}$, we can compute the linear combinations of the equation $P \stackrel{?}{=} h^{\mu} \cdot \mathbf{g}^{\mathbf{l}} \cdot \mathbf{h}^{\mathbf{r}}$ by using two valid transcripts and extracted the inner product argument witnesses for different x challenges.

If the extractor can compute a different representation of D or S with any other set of challenges (x, y, z), then this yields a non-trivial discrete logarithm relation between independent group elements $h, \mathbf{g}$ and $\mathbf{h}$, which contradicts the discrete logarithm assumption.

Then using these representations of D, S, $\mathbf{l}$ and $\mathbf{r}$, we find that for all challenges $x, y,$ and z

$$\mathbf{l} = \mathbf{d}z - \mathbf{y} + \mathbf{s}_L x \tag{25}$$

$$\mathbf{r} = \mathbf{d}z + \mathbf{y} + \mathbf{s}_R x \tag{26}$$

Once these equalities do not hold for $\mathbf{l}, \mathbf{r}$ and all challenges, then we have two distinct representations of the same group element using a set of independent group elements. This would be a non-trivial discrete logarithm relation.

Given y and z, we takes 3 transcripts for different x and use linear combinations of the equation $g^{\hat{t}}h^{\tau_x} \stackrel{?}{=} V^{z^2} g^{-\delta(y)} \cdot T_1^x \cdot T_2^{x^2} \in \mathbb{G}$ to compute τ_1, τ_2, t_1 and t_2 such that

$$T_1 = g^{t_1}h^{\tau_1}\ \wedge\ T_2 = g^{t_2}h^{\tau_2}. \tag{27}$$

Additionally we can compute a set of v, r such that $g^v h^r = V_1^{z^2}$, then we can compute (v_1, r_1) such that $g^{v_1} h^{r_1} = V_1$. If there exists any transcript $v_1 \cdot z^2 - \delta(y) + t_1 \cdot x + t_2 \cdot x^2 \neq \hat{t}$ then this directly yields a discrete log relation between g and h, i.e. a violation of the binding property of Pedersen commitment. If not, then for all y, z challenges and 3 distinct challenges $X = x_j, j \in \{1, 2, 3\}$:

$$\sum_{i=0}^{2} t_i X^i - \mathscr{P}(X) = 0 \tag{28}$$

with $t_0 = z^2 \cdot v_1 - \delta(y)$ and $\mathscr{P}(X) = \sum_{i=0}^{2} p_i \cdot X^i = \langle l(X), r(X) \rangle$. Since the polynomial $t(X) - \mathscr{P}(X)$ is of degree 2, but has at least 3 roots (each challenge x_j), it is necessarily the zero polynomial, i.e. $t(X) = \langle l(X), r(X) \rangle$.

Because this implies that $t_0 = p_0$, the following condition holds for all y, z challenges:

$$z^2 \cdot v_1 - \delta(y) =< z \cdot \mathbf{d}, z \cdot \mathbf{d} > + < z \cdot \mathbf{d}, \mathbf{y} > - < \mathbf{y}, z \cdot \mathbf{d} + \mathbf{y} > \in \mathbb{Z} \tag{29}$$

If this equality holds for n distinct y challenges and 3 distinct z challenges, then we can infer the following:

$$v_1 = \langle \mathbf{d}, \mathbf{d} \rangle \in \mathbb{Z}. \tag{30}$$

Because $g^{v_1} h^{r_1} = V_1$, we have that v_1 and r_1 are valid witnesses for the relation Eq 30. The extractor rewinds the prover $3 \cdot 3 \cdot n \cdot O(n^2)$ times. The extraction either returns a valid witness or a discrete logarithm relation between independently chosen group elements. In our definition, χ' is equal to χ but when χ extracts a discrete log relation, χ' fails. This would happen with at most negligible probability because of discrete log relation assumption. Therefore, we can use the forking lemma and see that the computational witness emulation holds.
